# Exploration of metformin as novel therapy for osteoarthritis: preventing cartilage degeneration and reducing pain behavior

**DOI:** 10.1186/s13075-020-2129-y

**Published:** 2020-02-22

**Authors:** Hui Li, Xiang Ding, Robert Terkeltaub, Hang Lin, Yuqing Zhang, Bin Zhou, Ke He, Kun Li, Zhichen Liu, Jie Wei, Yuanheng Yang, Hui Xie, Chao Zeng, Guanghua Lei

**Affiliations:** 1 Department of Orthopaedics, Xiangya Hospital, Central South University, 87 Xiangya Road, Changsha, 410008 Hunan China; 2 Department of Medicine, University of California at San Diego, San Diego, USA; 3 VA San Diego Medical Center, San Diego, USA; 4 Center for Cellular and Molecular Engineering, Department of Orthopaedic Surgery, University of Pittsburgh School of Medicine, Pittsburgh, USA; 5 Division of Rheumatology, Allergy, and Immunology, Department of Medicine, Massachusetts General Hospital, Harvard Medical School, Boston, USA; 6 The Mongan Institute, Massachusetts General Hospital, Harvard Medical School, Boston, USA; 7 Hunan Key Laboratory of Joint Degeneration and Injury, Changsha, China; 8 Department of Plastic and Cosmetic Surgery, Xiangya Hospital, Central South University, Changsha, China; 9 Movement System Injury and Repair Research Center, Xiangya Hospital, Central South University, Changsha, China; 10 Hunan Engineering Research Center of Osteoarthritis, Changsha, China; 11 National Clinical Research Center of Geriatric Disorders, Xiangya Hospital, Central South University, Changsha, China

**Keywords:** Osteoarthritis, Metformin, Mice, AMPK

## Abstract

**Background:**

Metformin could activate adenosine monophosphate-activated protein kinase (AMPK) which was postulated as a potential therapeutic target for osteoarthritis. This study aimed to examine the effects of metformin on cartilage and pain in osteoarthritis mouse model.

**Methods:**

Eighty 10-week-old male C57BL/6 mice were randomized to 6 groups: non-operation, sham-operation, destabilization of the medial meniscus (DMM)-operation with intragastric saline/metformin, and DMM-operation with intraarticular saline/metformin. Articular cartilage degeneration was examined by scanning electron microscopy (SEM) and graded using the scoring system recommended by Osteoarthritis Research Society International (OARSI). Mechanical withdrawal threshold and hind paw weight distribution were measured to assess the pain-related behavior. Cell Counting Kit-8 assay, quantificational real-time polymerase chain reaction, and western blot analysis were conducted to examine the anabolic and anti-catabolic effect of metformin and the role of AMPK in mediating its effects on interleukin-1β stimulated primary mice chondrocytes.

**Results:**

Compared with mice receiving intragastric and intraarticular saline, mice in both intragastric and intraarticular metformin displayed attenuated articular cartilage degeneration, indicated by less cartilage damage under SEM and significantly lower OARSI scores. A higher paw withdrawal threshold and a decreased weight-bearing asymmetry were observed in the intragastric and intraarticular metformin mice compared with their corresponding saline groups in DMM model of osteoarthritis. In vitro experiments showed that metformin not only decreased the level of matrix metalloproteinase 13, but also elevated type II collagen production through activating AMPK pathway.

**Conclusions:**

Metformin attenuates osteoarthritis structural worsening and modulates pain, suggesting its potential for osteoarthritis prevention or treatment.

## Background

Osteoarthritis (OA) is a disease characterized by articular cartilage degeneration and joint pain [1]. To date, there is no effective and safe treatment available that can halt OA progression [1]. Studies have shown that impaired mitochondrial biogenesis and function in articular chondrocytes were linked to OA [2–5], and activation of adenosine monophosphate-activated protein kinase (AMPK), a crucial cellular energy sensor [6], in chondrocytes promoted mitochondrial biogenesis and improved mitochondrial function in OA chondrocytes [7]. In vivo studies also reported that using non-selective AMPK activators such as berberine promoted both anti-catabolic and anti-apoptotic effects [8], whereas alpha1 subunit of AMPK (AMPKα1) knockout stimulated OA [9–11]. In addition, AMPK activation decreased the intensity of chronic pain by reducing the excitability of dorsal root ganglion neurons in inflammatory, post-surgical and neuropathic rodent model [12]. Thus, AMPK has been postulated as a potential therapeutic target for OA therapy [13–15].

The biguanide metformin has been used as a glucose-lowering medication for more than 60 years [16]. Moreover, increasing in vitro and in vivo evidence showed that metformin can delay aging and prolong lifespan [17, 18]. It has been reported that metformin acts via interference with mitochondrial respiratory complex I leading to a reduction in adenosine triphosphate (ATP) production [19], thereby activating AMPK [20]. In addition, metformin could activate AMPK via an adenine nucleotide-independent mechanism by stimulating phosphorylation of the Thr-172 on the alpha subunit of AMPK (AMPKα) [21]. However, to our knowledge, no in vivo study has been conducted to assess whether metformin could suppress OA progression and OA pain. To fill this knowledge gap, we examined the effect of intragastric and intraarticular metformin in an destabilization of the medial meniscus (DMM) model of OA in mice, known to cause destabilization of the joint which eventually leads to degeneration and pain of the joint, and investigated whether the anabolic and anti-catabolic effects of metformin on chondrocytes were mediated by the activation of AMPK.

## Methods

### Animals and experimental design

All experiments in this study were approved by the Committee on the Ethics of Animal Experiments of Xiangya Hospital, Central South University and carried out in strict accordance with the approved guidelines for the care and use of laboratory animals.

Eighty, 10-week-old, male C57BL/6 mice (mean weight: 27.3 g) were randomly assigned into 6 groups as the following:

- Non-operation group: no special treatment without operation.
- Sham-operation group: no special treatment with sham operation.
- Intragastric saline (IGS) group: normal saline (10 ml/kg) was administered intragastrically 3 days after the DMM surgery; once daily for 8 weeks.
- Intragastric metformin (IGM) group: metformin (200 mg/kg) was given 3 days after the DMM surgery; once daily for 8 weeks.
- Intraarticular saline (IAS) group: normal saline (1 ml/kg) was injected into the knee joint cavity 3 days after the DMM surgery; twice a week for 8 weeks.
- Intraarticular metformin (IAM) group: metformin (0.1 mmol/kg) was injected into the knee joint cavity 3 days after the DMM surgery; twice a week for 8 weeks.

The animals were housed in groups (four to five per cage) under controlled temperature on a 12-h light/dark cycle. Food and water were provided ad libitum.

### OA induction

After 1 week of acclimation, OA was induced by DMM as previously described [22]. Briefly, mice were anesthetized with intraperitoneal injection with 4% chloral hydrate (10 ml/kg body weight), and after being shaved and disinfected, the right knee joint was exposed through a medial parapatellar approach. The patella was dislocated laterally, and the knee was placed in full flexion followed by transection of anterior medial meniscotibial ligament with a microsurgical knife. Complete disruption of the ligament was confirmed visually by manually displacing the medial meniscus with fine forceps. The joint cavity was washed with normal saline solution. The articular capsule sutured with 6–0 absorbable PGA sutures, and the skin closed with 5–0 medical silk braided sutures.

Sham operation was performed on the right knee of a separate group of mice. It consisted of a skin incision and medial capsulotomy only, followed by capsule and skin closure as described above. Eleven mice died before the intervention ended. Among them, 3 mice died due to severe injury caused by fighting after DMM-operation (before intervention); 4 injured mice also caused by fighting were removed with euthanasia to prevent pain or stress (before intervention); 2 mice died of anesthetic accident (before intervention); and the rest two mice died with unknown reason after intervention (one in IGM group and the other in IAS group). At 8 weeks post-DMM surgery or sham operation, the remaining mice were euthanized with cervical dislocation after isoflurane anesthesia. Thirteen mice in non-operation group, 12 mice in sham-operation group, 11 mice in IGS group, 10 mice in IGM group, 12 mice in IAS group, and 11 mice in IAM group were included for further analysis.

### Scanning electron microscopy

Scanning electron microscopy (SEM) was performed to evaluate the surface ultrastructural characteristics of cartilage. After the mice were killed under anesthesia, the knee joints were isolated with scalpels and dissecting scissors and washed in 0.1 M phosphate buffer. Then the joints were fixed in 2.5% glutaraldehyde for 24 h and a second fixation step was performed with 1% osmic acid for 2 h. The specimens were washed in double-distilled water and dehydrated in a graded series of ethanol, then transferred into isoamyl acetate and dried with a critical point dryer (Hitachi High Technologies, Tokyo, Japan). The dried specimens were mounted on stages, coated with platinum/palladium (EiKO IB-5, Shawnee, USA) and observed using a HITACHI S-3400 N electron microscope (Hitachi High Technologies, Tokyo, Japan). We observed the alterations of the cartilage surface in the tibia regions.

### Histological analysis and OA scoring

Each dissected knee was fixed in 4% paraformaldehyde for over 24 h and decalcified in 15% EDTA, which was changed every 5 days for 20 days. The decalcified knee was dehydrated in a graded series of ethanol and embedded in paraffin (HistoCore Arcadia H, Leica, Nussloch, Germany). Serial frontal knee sections of the exact 5 μm thickness were obtained by using a Leica RM2255 microtome (Nussloch, Germany) across the entire knee joint. Then, the slices were stained with Safranin O/Fast Green to evaluate the entire articular cartilage of the knee. All images were taken using the same settings on a Nikon Eclipse Ti-S microscope (Melville, USA). Semi-quantitative histopathological scoring system recommended by Osteoarthritis Research Society International (OARSI) was performed for grading mouse cartilage degeneration (on a scale of 0–6) [23]. The severity of cartilage destruction was expressed as an average score of the three highest scores in all slides. The images were blinded-scored by two experienced scorers. If there was a disagreement on the score of cartilage destruction, the reading was adjudicated by a panel of three readers including the two who read the images. A consensus reading was reached when at least two of the three readers agreed.

### Pain-related behavior assessment

Mechanical allodynia and hind paw weight distribution were performed to assess pain-related behavior once a week from on day 0 (pre-operation) to day 56 post-surgery.

Mechanical allodynia was measured using an electronic von Frey anesthesiometer (IITC, Woodland Hills, CA, USA). Briefly, the plantar surface of the hind paw was stimulated with ascending force intensities of von Frey filaments. A brisk lifting of the foot was recorded as a positive response, and the number of positive responses for each stimulus was automatically recorded by the instrument. For each mouse, this test was performed three times with a time interval of 10 min between two adjacent stimuli. The mean value of the three readings was calculated as the final threshold value [24, 25].

Changes in hind paw weight distribution between the right (osteoarthritic) and left (control) limbs were measured as an index of joint discomfort in the osteoarthritic knee as previously described [26]. An incapacitance meter tester (IITC, Woodland Hills, CA, USA) was employed to evaluate hind paw weight distribution. Mice were placed in an angled plexiglass chamber positioned so that each hind paw rested on a separate force plate. The force exerted by each hind limb (measured in grams) is averaged over a 5-s period. Each data point is the mean of three, 5-s readings. The change in hind paw weight distribution was calculated by determining the difference in the amount of weight (g) between the left and right limbs.

To obtain consistent results, animals were allowed to adapt to the grid environment for 30 min. All behavioral tests were performed by the same technician who was blinded to the study groups and identification of animals in order to avoid subjective differences in interpretation, which could occur with different observers.

### Culture of articular chondrocytes and cartilage explants

To obtain mouse primary chondrocytes, we harvested the knee joints from the femoral condyles and tibial plateaus of postnatal day 3–4 C57BL/6 mice, and digested with 0.1% collagenase (Biosharp) overnight, as described previously [27]. A 2-mm biopsy punch was used to harvest macroscopically intact human cartilage explants from femoral condyles of total knee arthroplasty patients as described elsewhere [28]. Written informed consent was obtained from all participants.

### Treatments of chondrocytes and cartilage explants with metformin and AMPK inhibitor

Chondrocytes and cartilage explants were grown in culture medium with 10 ng/ml recombinant interleukin-1β (IL-1β) (R&D Systems, USA) and metformin (1, 10, and 20 mM, Sigma-Aldrich, USA). Chondrocytes and cartilage explants were also cultured in the presence of 10 ng/ml recombinant IL-1β alone. A control sample of chondrocytes and cartilage explants cultured in the absence of metformin and IL-1β was also evaluated. Finally, the effect of the addition of metformin was evaluated in the presence of 10 mM metformin and IL-1β samples, with or without dorsomorphin (10 uM, Sigma-Aldrich, USA), which is an AMPK inhibitor. Dimethyl sulfoxide (DMSO) was used as a vehicle of dorsomorphin. The chondrocytes in each group were respectively treated for 24 h by the corresponding intervention methods, then RNA and protein extraction were performed and the medium was collected. The cartilage explants were treated for 48 h. The medium was collected. All in vitro experiments and assays were repeated three times.

### Cell counting Kit-8 assay

The cell viability was assessed by Cell counting Kit-8 (CCK8) (Dojindo Laboratories, Kumamoto, Japan) according to the manufacturer’s protocol. The experiments were performed in sextuplicate.

### Total RNA extraction and quantificational real-time polymerase chain reaction

Total RNA was isolated using TRIzol reagent (Invitrogen). In brief, chondrocytes were washed with cold PBS and lysed directly in a dish by adding 1 ml of TRIzol reagent. After passing several times through a pipette, the homogenized samples were incubated for 5 min at room temperature, then transferred to a 1.5 ml RNase-free tube; 0.2 ml of chloroform was added to the lysate to extract RNA. The samples were centrifuged at 10,000×*g* for 15 min at 4 °C, and the upper aqueous phase was transferred into a fresh tube and mixed with 0.5 ml of isopropyl alcohol. Samples were incubated with ice-cold for 10 min and then centrifuged under 10,000×*g* for 10 min at 4 °C. After removing the supernatant, the RNA pellet was washed by adding 75% ethanol. The mixture was centrifuged under 10,000×*g* for 5 min at 4 °C before air-dry. The concentration of each sample was measured by NanoDrop 2000 (Thermo Scientific, USA). Complementary DNA (cDNA) synthesis was performed by 1 μg of total RNA using a cDNA synthesis kit (Trans Script, China) according to the manufacturer’s protocols. Gene expression assay primer pairs were ordered for the detection of matrix metalloproteinase 13 (*mmp13*) (primers: forward 5′-ACACTCAAATGGTCCCAAACG-3′, reverse 5′-TCATGATGTCAGCAGTGCCA-3′), type II collagen alpha 1 chain (*col2a1*) (primers: forward 5′-AGCGACTGTCCCTCGGAAAAAC-3′, reverse 5′-CCAGGTAGGCGATGCTGTTCTTAC-3′) and β-actin (primers: forward 5′- GGCTGTATTCCCCTCCATCG − 3′, reverse 5′- CCAGTTGGTAACAATGCCATGT − 3′). Quantitative analysis of the cDNA was performed using the ABI Quant Studio 3 (Applied Biosystems, USA) and All-in-one qPCR (Gene Copoecia, USA). The thermal cycling conditions were 95 °C for 10 min, 40 cycles of 95 °C for 15 s, 60 °C for 30 s, and 72 °C for 30 s. β-actin was used as the housekeeping gene for internal control. mRNA levels were normalized by β-actin levels of each sample. Comparative quantification was determined using the 2^−ΔΔCt^ method.

### Protein extraction and western blot

Cells were washed twice with ice-cold PBS and extracted by 2× SDS reagent with protease inhibitor cocktail (Roche, USA). After treatment with an ultrasonic cell disruption system, the cell lysate was clarified by centrifugation at 11,000 rpm for 10 min at room temperature, protein content in the supernatant was collected and the protein concentration was determined by BCA assay (Pierce, USA). Aliquots (30 μg) of protein were separated by 10% SDS-polyacrylamide gel electrophoresis and transferred onto a poly (vinylidene difluoride) membrane (Millipore, USA). The membrane was blocked with 5% (w/v) skimmed milk in TBST (10 mM Tris-HCl, pH 7.8, 150 mM NaCl, and 0.1% Tween-20) for 1 h and then incubated with anti-tubulin primary antibody (1:2000, Abcam, USA) or anti-GAPDH primary antibody (1:2000, Santa Cruz Biotechnology, USA), anti-MMP13 primary antibody (1:3000, Abcam, USA) or anti-type II collagen primary antibody (1:5000, Abcam, USA) or anti-phosphorylated alpha subunit of AMPK (pAMPKα) primary antibody (1:2000, Cell Signal Technology, USA) or anti-AMPK primary antibody (1:1000, Abcam, USA), in TBST containing 5% (w/v) BSA overnight at 4 °C. After washing three times, the blots were treated with anti-mouse and anti-rabbit IgG, respectively (1:5000, Cell Signal Technology, USA) in TBST containing 5% (w/v) BSA for 60 min, and the immune complex was detected using an ECL plus detection kit (Cell Signaling Technology, USA). Densitometric analysis was performed using ImageJ software (National Institutes of Health, USA).

### Enzyme-linked immunosorbent assay

Culture supernatant of chondrocytes and cartilage explants was collected after 24 h or 48 h of incubation respectively. The concentrations of MMP-13 were measured by enzyme-linked immunosorbent assay (ELISA) (mice chondrocytes: Cusabio, China; human cartilage explants: R&D Systems, UK) following the manufacturer’s instruction and were normalized to cell protein concentrations.

### Statistical analysis

All quantitative data were presented as means ± standard deviation (SD) and analyzed by Program Graph Pad Prism version 6.0. Multiple comparisons were performed by one-way ANOVA with Tukey’s post hoc test or repeated measures ANOVA with Bonferroni’s post hoc test as appropriate. The interaction effect between time and groups was also assessed in the repeated measures ANOVA. *p* value < 0.05 was considered statistically significant for all tests.

## Results

### Both intragastric and intraarticular metformin attenuated articular cartilage degradation in DMM-induced OA model

**Fig. 1:**
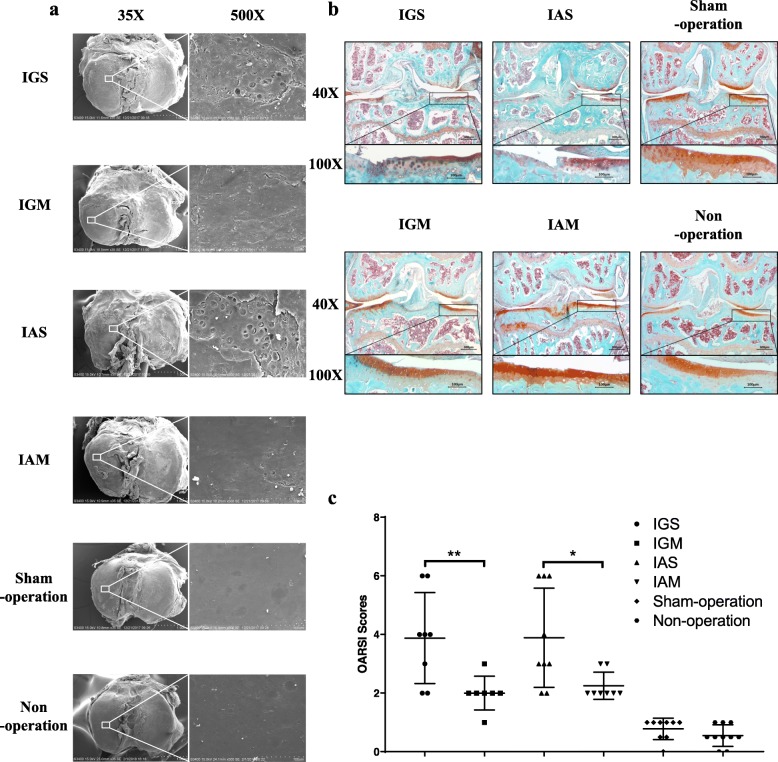
Effect of intragastric and intraarticular metformin on articular cartilage degradation in the destabilization of the medial meniscus (DMM)-induced OA mice model. **a** Representative scanning electron microscopy (SEM) images of the articular cartilage of the tibia plateau. Stripped cartilage, with a large area of exfoliation and exposed subchondral bone, was observed in the IGS and IAS groups. Mice in the IGM and IAM groups presented with slightly stripped cartilage and superficial avulsion. **b** Safranin O-fast green staining for frontal sections of knee at 8 weeks after surgery. The IGS and IAS groups presented with severe cartilage damage and less Safranin O staining. The IGM and IAM groups displayed a moderate degree of cartilage damage and loss of Safranin O staining. **c** Osteoarthritic changes in knee joints as quantified with the Osteoarthritis Research Society International (OARSI) score. Samples from the IGS and IAS groups showed more severe articular cartilage destruction compared with those from the IGM or IAM group. Data were expressed as the mean ± 95% confidence intervals. *p < 0.05; **p < 0.01. Statistical significance was calculated using one-way ANOVA with Tukey’s post hoc test. IGS, intragastric saline administration; IAS, intraarticular saline injection; IGM, intragastric metformin administration; IAM, intraarticular metformin injection

To investigate the ultrastructure of the cartilage surface at 8 weeks after surgery, SEM evaluation of the tibia plateau of surgical-induced OA mice was performed. As shown in Fig. 1a, the cartilage surfaces in mice in non-operation and sham-operated groups were smooth with no ultrastructure changes. Mice in either IGS group or in the IAS group had a large area of stripped cartilage and exfoliation and exposed subchondral bone with microcracks. A slightly stripped cartilage and superficial avulsion were shown in both the IGM and the IAM groups. In addition, the IGS and IAS groups presented with severe cartilage damage and less Safranin O staining, while the IGM and IAM groups displayed a moderate degree of cartilage damage and loss of Safranin O staining (Fig. 1b). The mean subjective scores recommended by OARSI among the IGS and IAS groups were both statistically significantly higher compared with that among the non-operation or sham-operation group; however, the OARSI score in the IGM group was statistically significantly lower than that in the IGS group (Fig. 1c). Similar results were observed between the IAM group and the IAS group.

### Both intragastric and intraarticular metformin modulated pain-related behavior in DMM-induced OA model

**Fig. 2:**
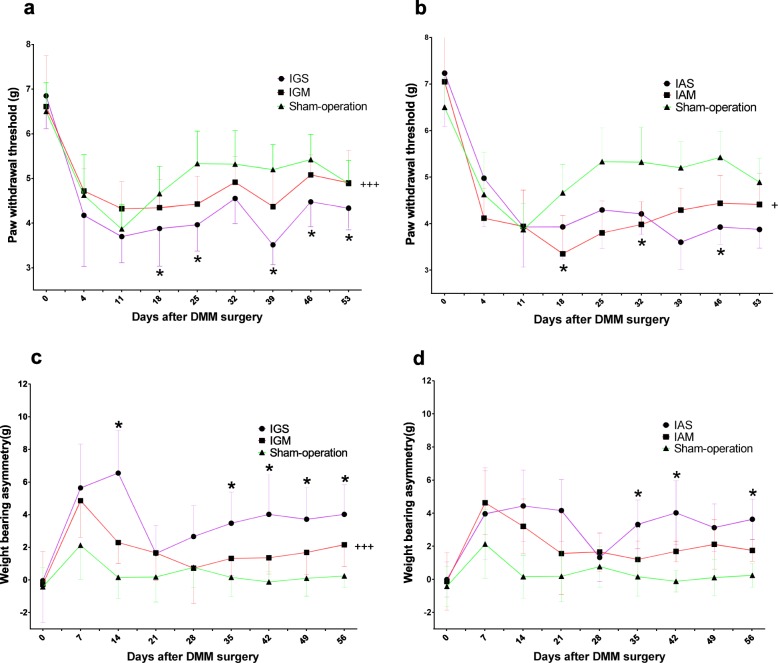
Effect of intragastric and intraarticular metformin on pain-related behavior in the destabilization of the medial meniscus (DMM)-induced OA mice model. Pain related behavior, measured as paw withdrawal thresholds to mechanical stimulation with a circular probe (**a**, **b**) or hindlimb weight-bearing asymmetry (**c**, **d**), increased after the induction of OA in mice by DMM surgery. Paw withdrawal threshold was higher in the IGM group than that in the IGS group (**a**). Similar results were observed between the IAM group and the IAS group (**b**). In addition, decreased weight-bearing asymmetry was observed in the IGM group compared with the IGS group (**c**). Difference with a possible trend toward significance (*p* = 0.052) in paw withdrawal threshold was observed among the IAM group and the IAS group (**d**). Data were expressed as the mean ± 95% confidence intervals. *p < 0.05, compared between the IGS and IGM groups or between the IAS and IAM groups at single time point, by repeated measures ANOVA with Bonferroni’s post hoc test; +*p* < 0.05; +++*p* < 0.001, compared between the IGS and IGM groups or between the IAS and IAM groups by repeated measures ANOVA with Bonferroni’s post hoc test; IGS, intragastric saline administration; IAS, intraarticular saline injection; IGM, intragastric metformin administration; IAM, intraarticular metformin injection

Mechanical hyperalgesia and hindlimb weight-bearing asymmetry were examined to assess the pain relief effect of IGM and IAM. The development of OA in the mice led to a decreased paw withdrawal thresholds and weight-bearing on the operated hindlimb, suggesting the occurrence of mechanical hyperalgesia and asymmetric of hindlimb weight-bearing (Fig. 2). Paw withdrawal threshold was higher in the IGM group than that in the IGS group (Fig. 2a). Similar results were observed between the IAM group and the IAS group (Fig. 2b). In addition, decreased weight-bearing asymmetry was observed in the IGM group compared with the IGS group. (Fig. 2c). Difference with a possible trend toward significance (*p* = 0.052) in paw withdrawal threshold was observed among the IAM group and the IAS group (Fig. 2d).

### Metformin protected against interleukin-1β-driven catabolism in chondrocytes and cartilage explants

**Fig. 3:**
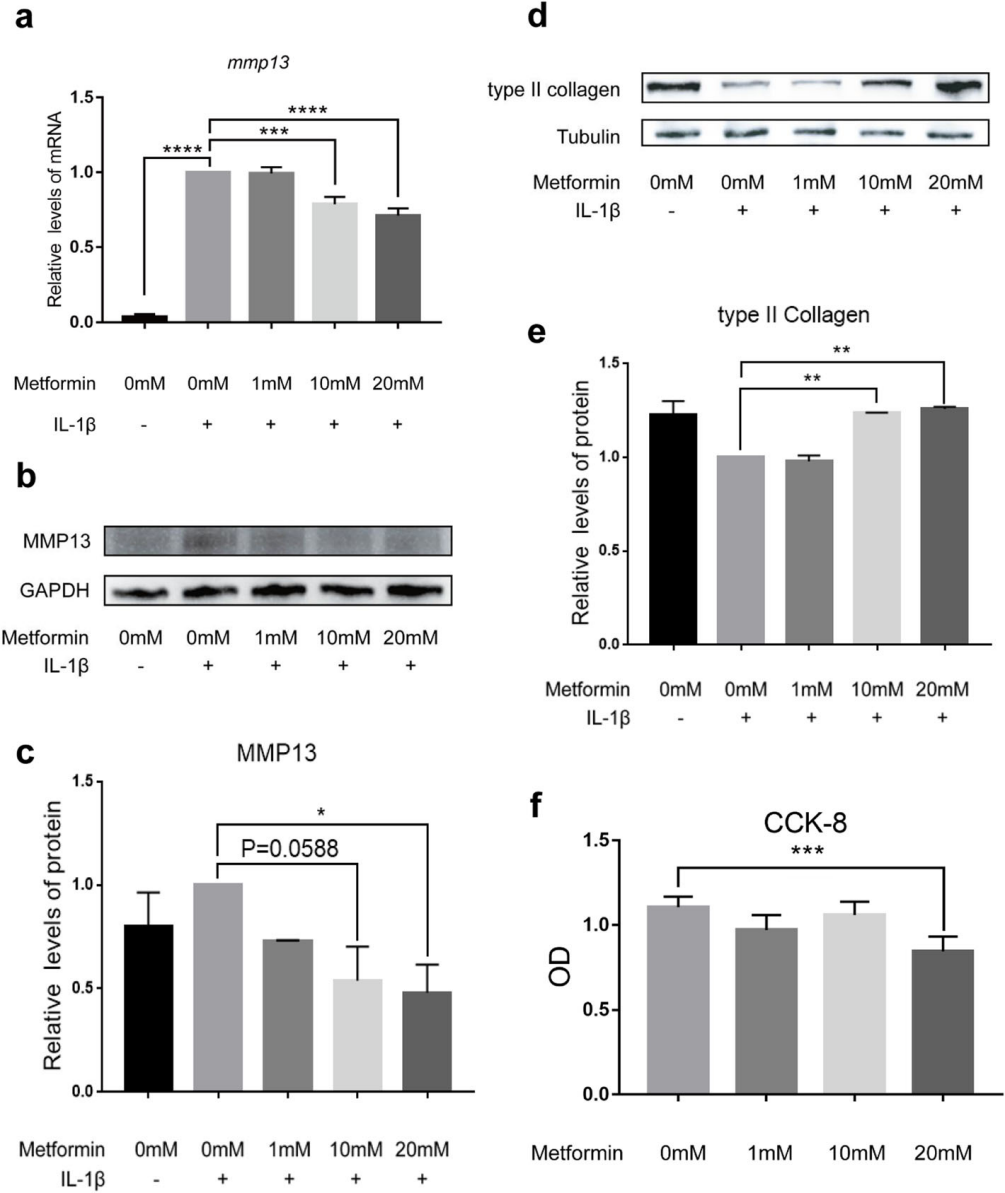
Metformin protected against interleukin-1β (IL-1β)-driven catabolism in chondrocytes. Mice articular chondrocytes (*n* = 3) were cultured with IL-1β and metformin (1, 10, and 20 mM) for 24 h, and the transcription of *mmp13* (**a**) was determined via qRT-PCR in mice articular chondrocytes treated with IL-1β and metformin (1, 10, and 20 mM). Protein levels of MMP13 (**b**) and type II collagen (**d**) were detected by western blot. The quantitation of protein expression of MMP13 (**c**) and type II collagen (**e**) was done by densitometry analysis of the protein bands. Values were normalized against tubulin or GAPDH. Chondrocyte viability was assessed with cell counting kit-8 (CCK8) assay (**f**). Data were expressed as the mean ± 95% confidence intervals. *p < 0.05; **p < 0.01; ***p < 0.001; ****p < 0.0001; Statistical significance was calculated using one-way ANOVA with Tukey’s post hoc test. MMP13, matrix metalloproteinase 13; OD, optical density; GAPDH, glyceraldehyde-phosphate dehydrogenase

To explore the underlying mechanism, we further examined whether metformin can protect against catabolism of interleukin-1β (IL-1β)-treated chondrocytes and cartilage explants in vitro. As shown in Fig. 3a, 24 h after metformin treatment the mRNA level of matrix metalloproteinase 13 (*mmp13*) in chondrocytes decreased in a dose-response manner. Such an effect was also shown by western blot (Fig. 3b, c). The expression levels of MMP13 in culture media of chondrocytes and cartilage explants were also decreased after metformin treatment (Additional file 1: Figure S1a-S1b). By contrast, metformin did not significantly modulate the mRNA levels of anabolic gene type II collagen alpha 1 chain (*col2a1*) (data not shown). Interestingly, 10 mM and 20 mM metformin significantly enhanced the expression level of type II collagen (Fig. 3d, e). Meanwhile, no statistically significant change of cell viability was found in chondrocytes treated with 1 mM or 10 mM metformin (Fig. 3f).

### AMPK activation was involved in the protective effect of metformin against IL-1β-driven catabolism in chondrocytes

**Fig. 4:**
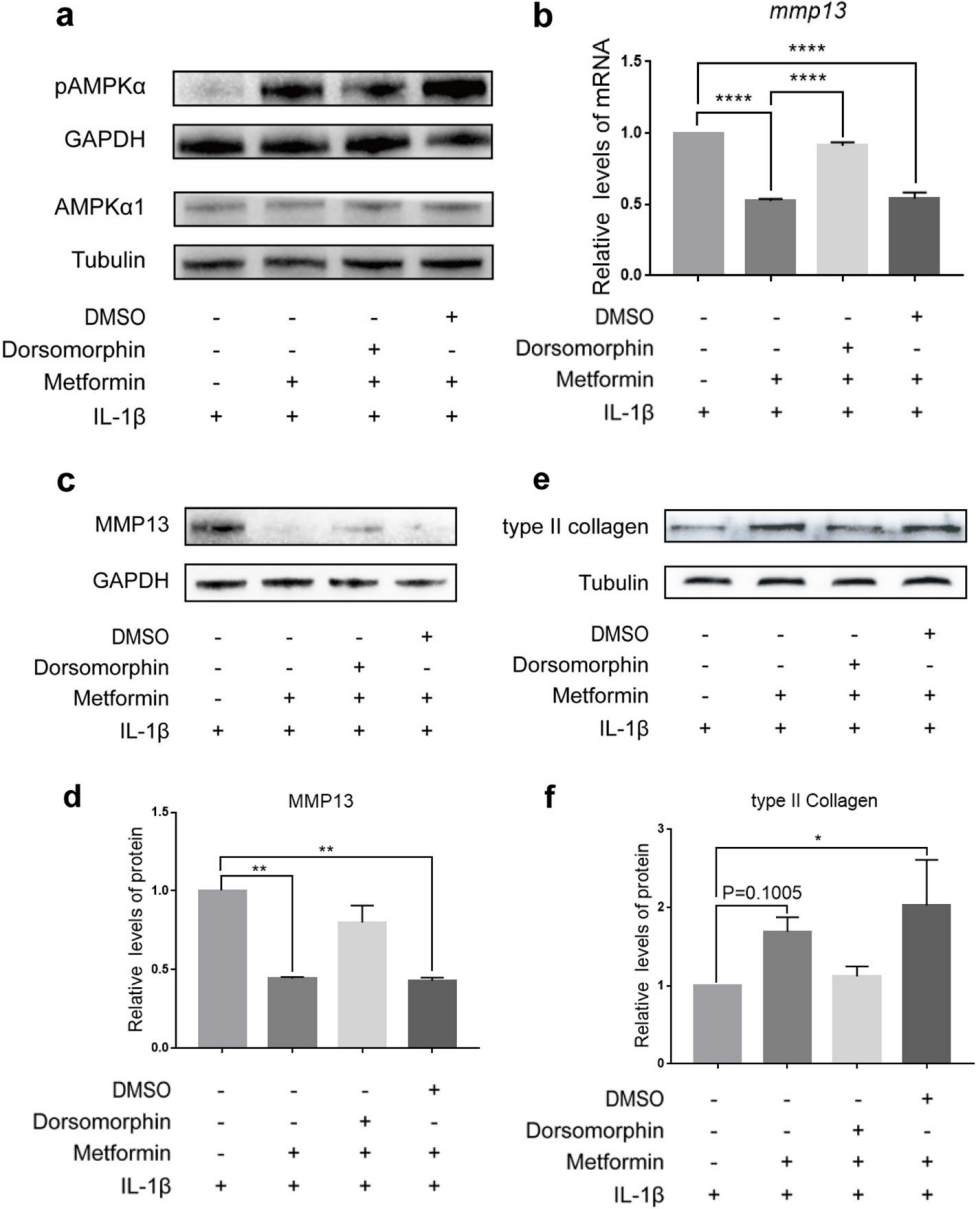
AMPK activation is involved in the protective effect of metformin against IL-1β-driven catabolism in chondrocytes. Mice articular chondrocytes (*n* = 3) were cultured in the absence of IL-1β, with or without 10 mM metformin, dorsomorphin, or dimethyl sulfoxide (DMSO), vehicle of dorsomorphin. Protein levels of pAMPKα and AMPKα1 (**a**) were detected by western blot. The transcription of *mmp13* (**b**) was determined by qRT-PCR. Protein levels of MMP13 (**c**) and type II collagen (**e**) were detected by western blot. The quantitation of protein expression of MMP13 (**d**) and type II collagen (**f**) was done by densitometry analysis of the protein bands. Values were normalized against tubulin or GAPDH. Data were expressed as the mean ± 95% confidence intervals. *p < 0.05; **p < 0.01; ***p < 0.001; ****p < 0.0001; statistical significance was calculated using one-way ANOVA with Tukey’s post hoc test. pAMPKα, phosphorylated alpha subunit of adenosine monophosphate-activated protein kinase; AMPKα1, alpha1 subunit of adenosine monophosphate-activated protein kinase; MMP13, matrix metalloproteinase 13; dimethylsulfoxide; GAPDH, glyceraldehyde-phosphate dehydrogenase

To clarify the mechanisms by which metformin led to decreased aggrecanase activity and proteoglycan breakdown by chondrocytes, we then investigated whether AMPK involved in the anti-catabolic effects of metformin. Ten millimolar metformin was selected to treat chondrocyte since it protects against catabolism without decreasing cell viability. There was no statistically significant change in the expression level of AMPKα1 in chondrocyte cultured in presence of IL-1β 24 h after treatment with metformin; however, the protein expression level of pAMPKα, indicating the activation of AMPK. Meanwhile, the effect of metformin on AMPKα1 was diminished when dorsomorphin, an inhibitor of AMPK, was added (Fig. 4a).

When dorsomorphin was added, the statistically significant higher expression level of *mmp13* was found in chondrocytes cultured in the presence of IL-1β, metformin, and dorsomorphin than chondrocytes cultured in presence of IL-1β and metformin. However, no difference was found when DMSO was added (Fig. 4b). Western blot analysis also showed that higher expression of MMP13 (Fig. 4c, d) but lower expression of type II collagen (Fig. 4e, f) were observed among chondrocytes when they were cultured in the presence of IL-1β, metformin, and dorsomorphin than those cultured in IL-1β and metformin. Moreover, the effects of metformin on expression of MMP13 or type II collagen were diminished when DMSO was added (Fig. 4c–f). Similar results were found in ELISA analysis measuring both MMP13 levels in culture media of chondrocytes and cartilage explants (Additional file 1: Figure S1c-S1d). These results indicated that the anti-catabolic effect of metformin was diminished when AMPK activation was inhibited.

## Discussion

In the present study, we found that both intragastric and intraarticular metformin attenuated articular cartilage degradation and modulated pain-related behavior in a DMM OA mice model, and metformin’s anabolic and anti-catabolic effects may be through its effect on the activation of AMPK. These findings provided new evidence of the potential therapeutic effect of metformin on OA.

### Comparison with previous studies

To date, there is a paucity of data regarding the effect of metformin on cartilage, chondrocyte, or pain in OA. A previous ex vivo study reported that metformin inhibited the release of NO, MMP3, and MMP13 of mice femoral head cartilage explants in response to IL-1β and TNF-α [11]. More recently, an in vitro study found that metformin suppressed IL-1beta-induced oxidative and osteoarthritis-like inflammatory changes [29]. In addition, a few observational studies have examined the relation of metformin use to the risk of OA, cartilage volume loss, or joint replacement; results, however, are conflicting. A cohort study of participants with OA and type 2 diabetes reported that patients receiving a combination of cyclooxygenase-2 inhibitors and metformin therapy had a lower risk of joint replacement than those receiving cyclooxygenase-2 inhibitors alone [30]. A more recent cohort study conducted among patients with radiographic knee OA and obese also showed that the rate of medial cartilage volume loss was lower in metformin users than non-users [31]. However, in another cohort study of patients with type 2 diabetes, no association was found between metformin prescription and the risk of OA, but no radiograph was available to confirm OA diagnosis [32]. In addition, all of three aforementioned studies did not use an active anti-diabetic medication as a comparator; thus, the findings may be susceptible to confounding by indication bias and the causal relationship between metformin and OA progression cannot be confirmed. A few studies also reported that metformin could prevent or reverse neuropathic pain by decreasing synaptic number, stimulating autophagy flux, and attenuating neuroinflammation [33–35].

### Possible explanations

While the biological mechanisms linking metformin to the attenuation of OA progression or pain relief are not fully understood, inhibition of AMPK may partly explain these findings. AMPK is an emerging regulator of the inflammatory process in OA [13–15]. Reduced AMPKα phosphorylation was noted in both the mice surgical instability-induced OA model and knee cartilage of human OA [10, 11]. AMPK deficiency in chondrocytes could disrupt articular cartilage homeostasis by enhancing catabolic activity and promoting chondrocyte apoptosis [20]. In addition, upregulating AMPK activity was showed to attenuate IL-1β and tumor necrosis factor-α induced catabolic gene expression in chondrocytes in vitro [10, 11]. Thus, the AMPK activator, i.e., metformin, may prevent the progression of OA. In the present study, AMPK inhibitor dorsomorphin inhibited anti-catabolic effect of metformin in chondrocyte, which indicated the involvement of AMPK pathway in the protective effect of metformin on cartilage.

In vivo animal studies have shown that either pharmacological activation or genetic regulation of AMPK had preventive, curative, and potential reversal effects on pain in models of nerve injury, chemotherapy-induced peripheral neuropathy, postsurgical pain, inflammatory pain, and diabetic neuropathy [12]. The underlying mechanisms involved the inhibition of signaling associated with pathological pain and reduction of dorsal root ganglia and trigeminal ganglion neuron excitability [12]. Thus, it could be speculated that metformin ameliorated OA-related pain behavior by modulating the AMPK signaling pathway as well.

### Limitations

First, in the mouse DMM model, metformin was given without a dose gradient, and identification and use of an optimal dosage might provide more useful information. Second, the role of AMPK in mediating chondroprotective effect of metformin was only measured in vitro, and further in vivo studies are warranted to verify this mechanism. Third, dorsomorphin was not a specific AMPK inhibitor which also inhibits BMP signaling and the VEGF type 2 receptor [36–38]. Notwithstanding its limitation, dorsomorphin was still used to inhibit AMPK in recent studies [39, 40] since it remains the only small molecule that has been found to inhabit AMPK signaling [41]. However, it would be more specific to apply AMPK knockout mouse models to examine the specific role of AMPK in mediating the chondroprotective and pain relief effects of metformin; thus, future studies are still warranted to explore. Finally, in the current study, we only assessed on potential mechanisms of metformin, i.e., activation of AMPK, further studies are needed to explore other pathways. Besides the AMPK-dependent effect, metformin may target multiple signaling pathways, e.g., mTOR, NF-κB, or inhibiting mitochondrial glycerophosphate dehydrogenase [42, 43].

## Conclusions

Metformin attenuates OA structural worsening, possibly through activating AMPK, and modulates pain, suggesting its potential for OA prevention or treatment.

## Supplementary information

**Additional file 1. **AMPK activation is involved in the effect of metformin on MMP13 of culture supernatant of chondrocytes and cartilage explants. Chondrocytes (a, c) and cartilage explants (b, d) were cultured in the absence of IL-1β, with or without 10 mM metformin, dorsomorphin or DMSO. Concentration of MMP13 of culture supernatant were detected by ELISA and were normalized to cell protein concentrations. Data were expressed as the mean ± 95% confidence intervals. * *p* < 0.05; ** *p* < 0.01; *** *p* < 0.001; MMP13, matrix metalloproteinase 13; DMSO, dimethylsulfoxide; IL-1β, interleukin-1β.

## Data Availability

The datasets analyzed during the current study are available from the corresponding author on reasonable request.

## Abbreviations

AMPK: Adenosine monophosphate-activated protein kinase
AMPKα: Alpha subunit of AMPK
AMPKα1: Alpha1 subunit of AMPK
ATP: Adenosine triphosphate
cDNA: Complementary DNA
col2a1: Type II collagen alpha 1 chain
DMM: Destabilization of the medial meniscus
DMSO: Dimethyl sulfoxide
IAM: Intraarticular metformin
IAS: Intraarticular saline
IGM: Intragastric metformin
IGS: Intragastric saline
IL-1β: Interleukin-1β
mmp13: Matrix metalloproteinase 13
OA: Osteoarthritis
OARSI: Osteoarthritis Research Society International
pAMPKα: Phosphorylated alpha subunit of AMPK
SD: Standard deviation
SEM: Scanning electron microscopy
