# Supplementary material for: Exploration of metformin as novel therapy for osteoarthritis: preventing cartilage degeneration and reducing pain behavior
Source: Arthritis Res Ther. 2020 Feb 22;22:34. doi: 10.1186/s13075-020-2129-y (PMC7036179; doi:10.1186/s13075-020-2129-y)
Supplement: Supplementary file 1 — Additional file 1. AMPK activation is involved in the effect of metformin on MMP13 of culture supernatant of chondrocytes and cartilage explants. Chondrocytes (a, c) and cartilage explants (b, d) were cultured in the absence of IL-1β, with or without 10 mM metformin, dorsomorphin or DMSO. Concentration of MMP13 of culture supernatant were detected by ELISA and were normalized to cell protein concentrations. Data were expressed as the mean ± 95% confidence intervals. * p < 0.05; ** p < 0.01; *** p < 0.001; MMP13, matrix metalloproteinase 13; DMSO, dimethylsulfoxide; IL-1β, interleukin-1β. [file 13075_2020_2129_MOESM1_ESM.pdf]

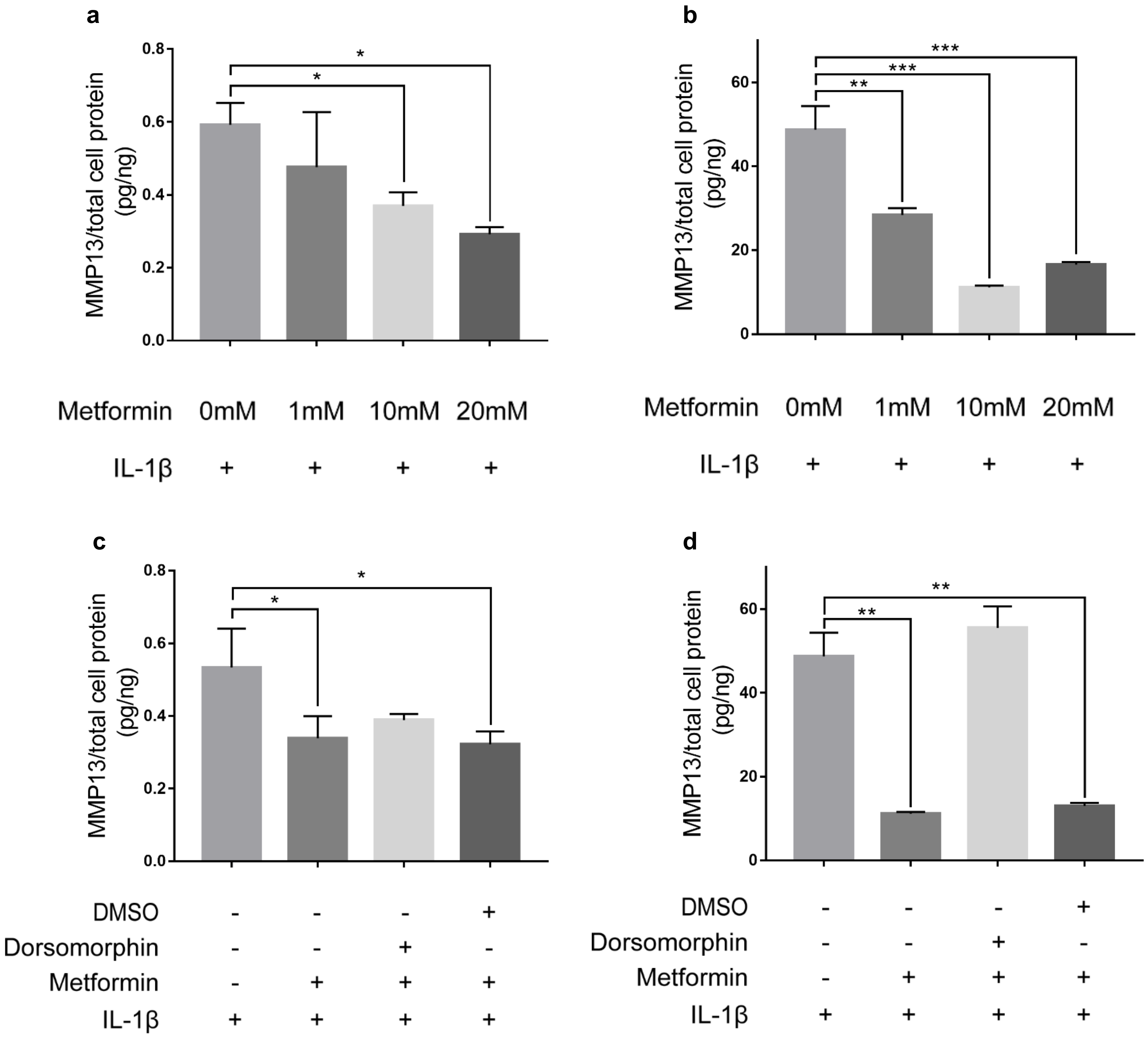
a
MMP13/total cell protein (pg/ng)
0.0
0.2
0.4
0.6
0.8
*
*
Metformin 0mM 1mM 10mM 20mM
IL-1β + + + +
b
MMP13/total cell protein (pg/ng)
0
20
40
60
**
***
***
Metformin 0mM 1mM 10mM 20mM
IL-1β + + + +
c
MMP13/total cell protein (pg/ng)
0.0
0.2
0.4
0.6
0.8
*
*
DMSO - - - +
Dorsomorphin - - + -
Metformin - + + +
IL-1β + + + +
d
MMP13/total cell protein (pg/ng)
0
20
40
60
**
**
DMSO - - - +
Dorsomorphin - - + -
Metformin - + + +
IL-1β + + + +
